# Making the Public Understand

**Published:** 1920-08-28

**Authors:** 


### THE EDITOR'S LETTER-BOX.

**Correspondence on all subjects is invited, but we cannot in any way be responsible for the opinions expressed by our correspondents, who must give their name and address as a guarantee of good faith, but not necessarily for publication. Correspondents are reminded that brevity of style and conciseness of statement greatly facilitate early insertion.**

### Making the Public Understand.

*To the Editor of* THE HOSPITAL.

SIR,—That excellent little article "The Way of Attainment," written by a matron, a member of the College of Nursing, expresses my views so admirably that I trust she will forgive me if I venture to make one comment. That is, Who are our Leaders—in the profession? One naturally inclines to think those who represent us on (*a*) The Council of the College of Nursing, (*b*) The General Nursing Council. The latter have now decided to admit Press representatives to their meetings. It would, I am confident, be intensely interesting both to the 18,000 members of the College, and at least to a section of the reading public, if we were honoured with a report of the College monthly meeting in the press and nursing press. This would effect a threefold purpose: (1) To instruct the general public in professional matters; (2) To get the full benefit of public opinion; (3) To keep members in touch with the trend of its progress and to encourage their opinions.

Although we have every confidence in our representatives, it would appear we have arrived at a period of time when no section of the community can any longer balance themselves on the knife-edge of absolute indifference to the general trend of public opinion, and more especially if that body is representing 18,000 members or so. Perhaps the *Bulletin* has been originated to meet this want, but with all due respect to the *Bulletin* and its literary staff, it would be preferable to have the press and nursing press, by the medium of which we would not only "wake up" a slumbering public, but encourage an exchange of professional opinions and get more in touch with the isolated members, and in cases where help is required it could be brought forward. Individual action is of little use nowadays. To be effective it must have a united voice, and there appears no reason why the press and nursing press could not become the organ of expression. Perhaps "Our Leaders" will give the matter their kind consideration and prompt attention.

There is always a long period of antenatal growth before the new idea, the new way of looking at things, can come to birth. A COLLEGE MEMBER.

